# Induced accumulation of serotonin in gibberellin A_3_-treated suspension cells of giant bamboo (*Dendrocalamus giganteus*)

**DOI:** 10.5511/plantbiotechnology.25.0113a

**Published:** 2025-03-25

**Authors:** Taiji Nomura, Shinjiro Ogita, Yasuo Kato

**Affiliations:** 1 Biotechnology Research Center and Department of Biotechnology, Toyama Prefectural University, 5180 Kurokawa, Imizu, Toyama 939-0398, Japan; 2 Department of Development of Local Resources, Faculty of Bioresource Sciences, Prefectural University of Hiroshima, 5562 Nanatsukacho, Shobara, Hiroshima 727-0023, Japan

**Keywords:** bamboo cells, *Dendrocalamus*, 5-hydroxytryptamine, gibberellin A_3_, serotonin

## Abstract

Rational metabolic-flow switching is an effective strategy that we previously proposed to produce exogenous high-value secondary metabolite(s) in cultured plant cells. Specifically, it involves redirecting a highly active inherent metabolic pathway to a pathway producing related exogenous compounds. The success of this strategy depends on the identification of at least one highly active metabolic pathway in host plant cells that can be redirected to produce a target compound following the introduction of exogenous biosynthetic gene(s) via genetic transformation. Active metabolic pathways may be predicted on the basis of the major metabolites that accumulate in cells. In previous proof-of-concept studies, we demonstrated that cultured cells of a temperate bamboo species (*Phyllostachys nigra*; Pn) are an appropriate host for producing phenylpropanoid-derived compounds. However, developing a series of host plant cells with a variety of metabolic properties is necessary to maximize the utility of rational metabolic-flow switching. In this study, we established cultured cells of two tropical bamboo species (*Dendrocalamus giganteus* and *Dendrocalamus brandisii*). By analyzing the metabolites that increased in abundance in response to phytohormone treatments, we determined that exogenous gibberellin A_3_ (GA_3_) substantially induced the accumulation of an unknown metabolite in *D. giganteus* (Dg) cells. This compound was isolated and identified as serotonin (5-hydroxytryptamine). After optimizing the culture conditions, the serotonin production titer in Dg suspension cells reached 360 mg l^−1^. These findings indicate that Dg cells are potentially suitable for the bioproduction of exogenous tryptophan-derived indolic compounds via rational metabolic-flow switching.

## Introduction

Plants produce a diverse array of secondary metabolites, which are also known as specialized metabolites or natural products. There are an estimated 200,000–1,000,000 plant secondary metabolites (Afendi et al. 2012; Saito and Matsuda 2010), of which many have received considerable attention because they are sources of industrial and medicinal materials for human use (Balandrin et al. 1985; Hines and Zahn 2012). The commercial production of secondary metabolites from plant resources can be limited by environmental factors and the long period required for plant growth and accumulation of metabolites to the desired concentration (Chandran et al. 2020; Marchev et al. 2020). Such limitations may be overcome by using cultured plant cells. For example, cultured plant cells are useful for producing a target compound that is originally present in the mother plant (Wilson and Roberts 2012). This approach, which does not require transgenic plant cells, has been used for the industrial production of shikonin, the naphthoquinone pigment with anti-inflammatory activity in *Lithospermum erythrorhizon*, in *L. erythrorhizon* cultured cells (Yazaki 2017). Cultured plant cells may also be used to produce a target compound that is not originally present in the mother plant, which requires the creation of transgenic plant cells. Although heterologous production of plant secondary metabolites in microbial hosts has been pursued extensively, the use of cultured plant cells has been limited by certain factors that are not applicable to microbes, including a long culture period and laborious transformation processes. Nevertheless, plant cells are typically the most suitable hosts for the heterologous expression of exogenous plant enzyme-encoding genes, with most of the obstacles associated with heterologous expression in microbial hosts likely avoided by using cultured plant cells.

To efficiently produce “exogenous” secondary metabolites in transgenic cultured plant cells, we previously developed a “rational metabolic-flow switching strategy” (Nomura et al. 2018); “exogenous” metabolite refers to a compound that is not inherently present in the wild-type host cells, but is produced in transgenic cells expressing the biosynthetic gene(s) from other organisms. The first step of this procedure is determining the major metabolite(s) in the plant cells of interest to identify highly active metabolic pathway(s). The second step involves transforming cells with exogenous gene(s) encoding the enzyme(s) required to switch the inherent active metabolic flow to the biosynthesis of the exogenous target compound(s). Cultured cells of *Phyllostachys nigra* (Pn), a temperate bamboo species, were used to demonstrate the effectiveness of this strategy because they proliferate efficiently in liquid medium and accumulate substantial amounts of phenylpropanoid-derived metabolites, such as hydroxycinnamoylputrescines (e.g., feruloylputrescine and *p*-coumaroylputrescine) and lignin, depending on culture conditions (Nomura et al. 2013; Ogita 2005; Ogita et al. 2012). We generated transgenic Pn cells expressing the barley (*Hordeum vulgare*) agmatine coumaroyltransferase gene (*HvACT*) (Nomura et al. 2018). An analysis of these *HvACT*-transformed Pn cells revealed that *p*-coumaroylagmatine and feruloylagmatine were produced efficiently, while the accumulation of feruloylputrescine and *p*-coumaroylputrescine decreased substantially, reflecting a successful biosynthetic pathway switch from the production of putrescine amides to the production of agmatine amides. We also achieved metabolic-flow switching by expressing exogenous genes of bacterial origin in Pn cells. Transgenic Pn cells expressing the *Pseudomonas putida* KT2440 4-hydroxycinnamoyl-CoA hydratase/lyase gene (*PpHCHL*) produced mono- and/or di-glucose conjugates of 4-hydroxybenzoic acid and vanillic acid (Kitaoka et al. 2020; Ube et al. 2024), whereas those expressing the *Bacillus amyloliquefaciens* phenolic acid decarboxylase gene (*BaPAD*) produced primeverose conjugates of 4-vinylphenol and 4-vinylguaiacol (Kitaoka et al. 2021). The highest production titer obtained to date is 1.7 g l^−1^ for 4-hydroxybenzoic acid glucose ester in *PpHCHL*-transformed Pn cells. These three proof-of-concept studies demonstrated the efficacy of the rational metabolic-flow switching strategy for the production of exogenous metabolites as well as the suitability of Pn cells for producing phenylpropanoid-derived metabolites.

To increase the versatility of rational metabolic-flow switching, a series of host plant cells and culture conditions with a variety of metabolic properties should be developed, especially those relevant to metabolites other than phenylpropanoids, which can be produced in Pn cells. Various types of exogenous secondary metabolites, such as alkaloids and terpenoids, may then be produced in transgenic cultured plant cells via rational metabolic-flow switching. In this study, we established cultured cells of two tropical bamboo species belonging to the genus *Dendrocalamus*. By examining metabolic properties following phytohormone treatments, we revealed the strong inductive effects of gibberellin A_3_ (GA_3_) on one compound in *Dendrocalamus giganteus* (Dg) cells. The GA_3_-induced compound was isolated and structurally characterized. Moreover, culture conditions were optimized to enhance the production of the GA_3_-induced compound in Dg cells. On the basis of the study results, the potential utility of the metabolic activities of Dg cells for increasing the versatility of the rational metabolic-flow switching strategy is discussed.

## Materials and methods

### Chemicals

Indole-3-acetic acid, 2,4-dichlorophenoxyacetic acid, 6-benzyladenine, *trans*-zeatin, thidiazuron, methyl jasmonate, salicylic acid, and GA_3_ were purchased from Fujifilm Wako Pure Chemical Corporation (Osaka, Japan). Picloram, abscisic acid, and authentic serotonin (5-hydroxytryptamine hydrochloride) were purchased from Tokyo Chemical Industry (Tokyo, Japan).

### NMR, UV, and MS analyses

NMR spectra were recorded using an AVANCE 400 spectrometer (Bruker, Karlsruhe, Germany), with D_2_O serving as the solvent. UV spectra were measured using a UV-1800 spectrophotometer (Shimadzu, Kyoto, Japan), with H_2_O serving as the solvent. High-resolution electrospray ionization time-of-flight mass spectrometry (HR-ESI-TOF-MS) spectra were obtained using a micrOTOF focus spectrometer (Bruker) by a previously described direct infusion method (Nomura and Kato 2020).

### Generation of cultured cells

Edible bamboo shoots were collected from Dg plants at the Botanic Gardens of Toyama (Toyama, Japan) and from *Dendrocalamus brandisii* (Db) plants at the Fuji Bamboo Garden (Nagaizumi-cho, Sunto-gun, Shizuoka) (Supplementaty Figure S1). Approximately 10–20 cm long young shoots were used to generate calli as previously described (Ogita 2005). Briefly, sterilized shoot segments were cultured on a half-strength (1/2) Murashige and Skoog medium (Murashige and Skoog 1962) supplemented with 3 µM 2,4-dichlorophenoxyacetic acid and 3% (w/v) sucrose in darkness at 25°C. In the first culture phase (2–4 weeks), some shoot segment parts enlarged and produced a few whitish-yellow calli. In the second phase (i.e., subcultures; 2–6 months), the necrotic portions of shoot segments and calli were removed to select uniform cell lines (Supplementaty Figure S2). Suspension cultures were generated from the uniform calli as previously described (Ogita et al. 2011).

### Subculture and phytohormone treatment of cultured cells

Suspension cells were maintained in modified Murashige and Skoog (mMS) liquid medium supplemented with 680 mg l^−1^ KH_2_PO_4_, 10 µM picloram, and 3% (w/v) sucrose (Ogita 2005; Ogita et al. 2011), which strongly promotes cell proliferation. Cells were subcultured in 100 ml liquid medium in a 300 ml Erlenmeyer flask that was maintained on a rotary shaker (100 rpm) in darkness at 25°C. Cells were subcultured every 2 weeks by adjusting the initial sedimented cell volume (SCV) to 5% as previously described (Ogita et al. 2011). Callus cells were maintained on mMS medium solidified with 0.3% (w/v) gellan gum in a Petri dish (φ=90 mm). Cultures were incubated in darkness at 25°C and subcultured every 3 weeks by transferring the calli [approximately 100 mg fresh weight (FW)] to fresh medium.

For the phytohormone treatment of callus cells, 3-week-old subcultured calli were transferred to 1/2 MS solid medium containing a phytohormone at 10 µM and cultured in darkness at 25°C for 2 weeks. The phytohormones used for treatments were auxins (indole-3-acetic acid and 2,4-dichlorophenoxyacetic acid), cytokinins (6-benzyladenine, *trans*-zeatin, and thidiazuron), and others (GA_3_, abscisic acid, methyl jasmonate, and salicylic acid). After 2 weeks, calli were collected and stored at −30°C until used.

For the GA_3_ treatment of Dg suspension cells, 2-week-old subcultured suspension cells were transferred to 1/2 MS liquid medium containing 0.1–10 µM GA_3_, with an initial cell density of 5% or 10% SCV, and then cultured for 14 or 30 days under the same conditions as described above. Cells were collected every 2 or 3 days as previously described (Nomura et al. 2013) and stored at −30°C until used.

### Extraction and HPLC analysis

Cells (approximately 100 mg FW) were mixed with 10 volumes of 50% (v/v) MeOH containing 2% (v/v) AcOH for a 10-min ultrasonication at room temperature. The resulting extract was centrifuged (21,500×g, 10 min, 4°C). The supernatant was diluted with water (1 : 5) and then analyzed using a reversed-phase HPLC system (column, Mightysil RP-18 GP Aqua, 5 µm, 4.6×150 mm; Kanto Chemical, Tokyo, Japan; solvent, 10% (v/v) acetonitrile containing 0.1% (v/v) trifluoroacetic acid; flow rate, 0.8 ml min^−1^; detection wavelength, 280 nm).

### Isolation and structural characterization of GA_3_-induced compound 1 from Dg suspension cells

Dg suspension cells were cultured for 21 days in 1/2 MS medium supplemented with 1 µM GA_3_, with an initial cell density of 10% SCV. Cells from a 1-l culture (100 ml ×10) were collected on filter paper via vacuum filtration. The collected cells (93 g FW) were mixed with 1 l 50% (v/v) MeOH for a 30-min ultrasonication at room temperature. The resulting extract was filtered through a diatomite pad (Radiolite #3000; Showa Chemical Industry, Tokyo, Japan), which was used as a filtration aid, placed on a filter paper in a Büchner funnel. The filtrate was concentrated and defatted by washing three times with *n*-hexane. The resulting aqueous layer was concentrated and dissolved in 30 ml water. After adjusting the pH to 7 with 1 M NaOH, the solution was applied to an octadecylsilyl (ODS) column (Cosmosil 75C18-OPN; Nacalai Tesque, Kyoto, Japan; 4×32 cm; 400 ml column volume) equilibrated with water. The following solvents (2 l each) were used for the sequential elution from the column: water, 40% (v/v) MeOH, and 40% (v/v) MeOH containing 0.1% (v/v) AcOH. The eluate derived from the elution with 40% MeOH containing 0.1% AcOH was concentrated to a small volume, passed through a membrane filter (Millex-HV, 0.45 µm; Merck, Darmstadt, Germany), and subjected to reversed-phase preparative HPLC (column, TSKgel ODS-80Ts; 5 µm, 20×250 mm; Tosoh, Tokyo, Japan; solvent, 10% (v/v) MeOH containing 0.1% (v/v) AcOH; flow rate, 5 ml min^−1^; detection wavelength, 280 nm). The collected fraction was concentrated and lyophilized to obtain compound 1 (246 mg pale yellow powder).

The spectral properties of serotonin (5-hydroxytryptamine, 1) (acetic acid salt) were as follows: HR-ESI-TOF-MS (Supplementary Figure S3) (positive) *m*/*z* (relative intensity) 177.1028 [M+H]^+^ (53) (calcd for C_10_H_13_N_2_O^+^, 177.1022), *m*/*z* 160.0803 [M-NH_2_]^+^ (100); UV (H_2_O) λ_max_ (logε) 275 nm (3.69); ^1^H-NMR (400 MHz, D_2_O, Supplementary Figure S4A): δ (ppm; relative to HOD set to 4.80) 3.07 (2H, t, *J*=7.0 Hz, H-8), 3.27 (2H, t, *J*=7.0 Hz, H-9), 6.85 (1H, dd, *J*=8.8, 2.4 Hz, H-6), 7.06 (1H, d, *J*=2.4 Hz, H-4), 7.25 (1H, s, H-2), 7.38 (1H, d, *J*=8.8 Hz, H-7); ^13^C-NMR (100 MHz, D_2_O, Supplementary Figure S4B): δ (ppm) 22.5 (C-8), 39.5 (C-9), 102.4 (C-4), 108.3 (C-3), 111.8 (C-6), 112.8 (C-7), 125.2 (C-2), 127.0 (C-3a), 131.6 (C-7a), 148.7 (C-5).

## Results

### Analysis of metabolites in Dg and Db cultured cells

**Figure 1:**
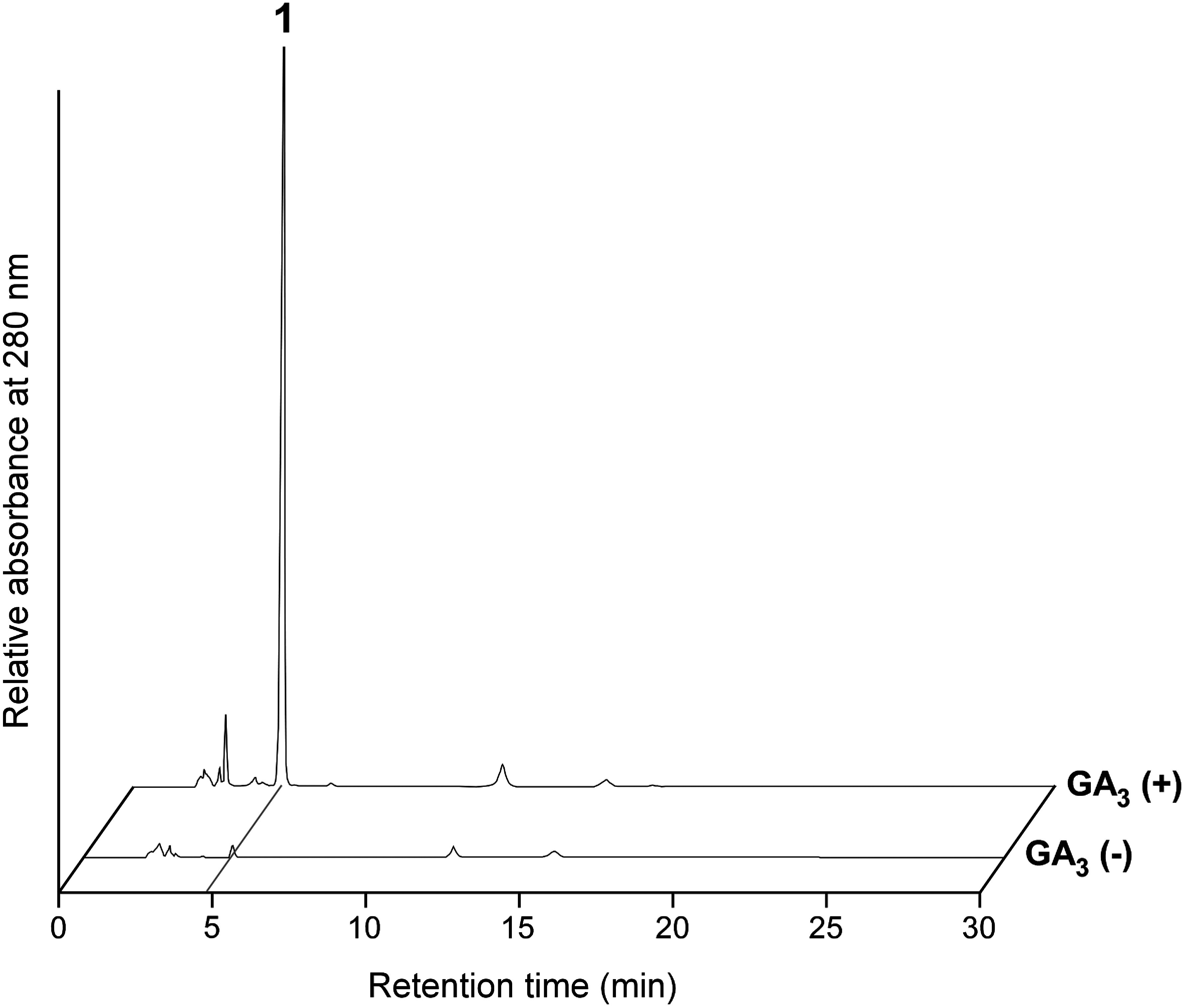
Figure 1. HPLC chromatograms showing the induced accumulation of compound 1 in Dg cells treated with GA_3_. Extracts of 2-week-old cells cultured in the presence (+) or absence (−) of 10 µM GA_3_ were analyzed.

We generated Dg and Db cultured cells from young edible bamboo shoots. Because of the active proliferation of callus cells on solid mMS medium, cells were subcultured every 3 weeks. For the phytohormone treatments of calli, 1/2 MS was used as a basal medium because mMS medium promotes cell proliferation, but tends to suppress secondary metabolism (Nomura et al. 2013, 2021; Ogita et al. 2012). Calli for both species were treated with nine phytohormones (10 µM). Cells were collected 2 weeks after starting the treatment, after which methanolic cell extracts were analyzed by reversed-phase HPLC. A 10-fold increase in the HPLC peak following a phytohormone treatment (relative to the peak for the non-treated control) was set as the criterion for detecting a phytohormone-induced compound. Accordingly, the HPLC peak at a retention time of 4.8 min for GA_3_-treated Dg cells was considered to correspond to a phytohormone-induced compound (hereafter compound 1; Figure 1). None of the other combinations of calli and phytohormones resulted in the substantial induction of metabolite production, although some compounds that were weakly induced in response to phytohormones were detected.

### Identification of serotonin as a GA_3_-induced secondary metabolite in Dg suspension cells

We purified compound 1 from 21-day-old Dg suspension cells (93 g FW) cultured in 1/2 MS medium supplemented with 1 µM GA_3_. During our first attempt to purify this compound, we applied the concentrated acidic cell extract to an ODS column equilibrated with acidic water, but compound 1 passed through the column, suggesting it is a basic compound. Thus, for our second attempt, the concentrated cell extract was neutralized and then applied to a column that had been equilibrated with water lacking acetic acid. Under this condition, compound 1 was retained in the column and then eluted using 40% MeOH containing 0.1% acetic acid. The subsequent purification via preparative HPLC resulted in 246 mg of compound 1.

**Figure 2:**
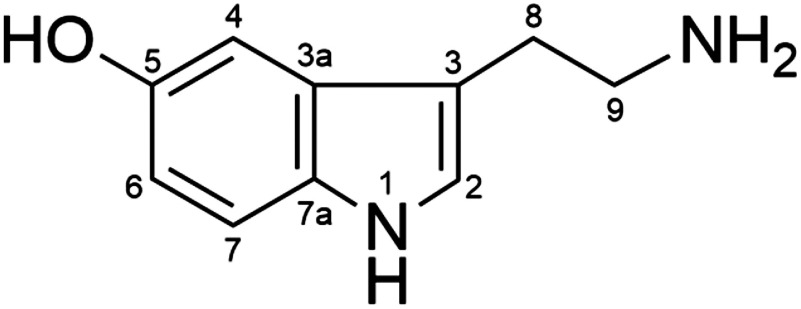
Figure 2. Chemical structure of serotonin (5-hydroxytryptamine).

The molecular formula of compound 1 was C_10_H_12_N_2_O as determined by HR-ESI-TOF-MS (*m*/*z* 177.1028 [M+H]^+^, Supplementary Figure S3). The mass spectrum of compound 1 had a characteristic fragment ion at *m*/*z* 160.0803 corresponding to the deaminated form [M-NH_2_]^+^. The presence of a primary amino group in compound 1 was confirmed by a colorimetric analysis using a ninhydrin reagent (Supplementary Figure S5A). In the ^1^H-NMR spectrum (Supplementary Figure S4A), three coupled aromatic signals at 7.38 (H-7), 7.06 (H-4), and 6.85 (H-6) ppm indicated that compound 1 has a 1,2,4-trisubstituted benzene ring. The presence of another aromatic proton detected as a singlet signal at 7.25 ppm, two coupled methylene groups detected as triplet signals (3.07 and 3.27 ppm), and one nitrogen atom other than the primary amino group suggested that compound 1 is a tryptamine derivative with an indole skeleton as the aromatic moiety; this was confirmed by a colorimetric analysis using Ehrlich’s reagent (Supplementary Figure S5B). According to the molecular formula, the remainder of the structure comprised one hydroxy group; the presence of a phenolic hydroxy group was confirmed by a colorimetric analysis using FeCl_3_ (Supplementary Figure S5C). Therefore, compound 1 was identified as serotonin (5-hydroxytryptamine, Figure 2). The spectral data were in accordance with relevant published data (Salmoun et al. 2002; Watanabe 1999). The identity of compound 1 was also verified by comparing the HPLC retention time, HR-ESI-TOF-MS spectrum, and ^1^H- and ^13^C-NMR spectra with those of authentic serotonin.

### Effects of GA_3_ dosing conditions on serotonin production

**Figure 3:**
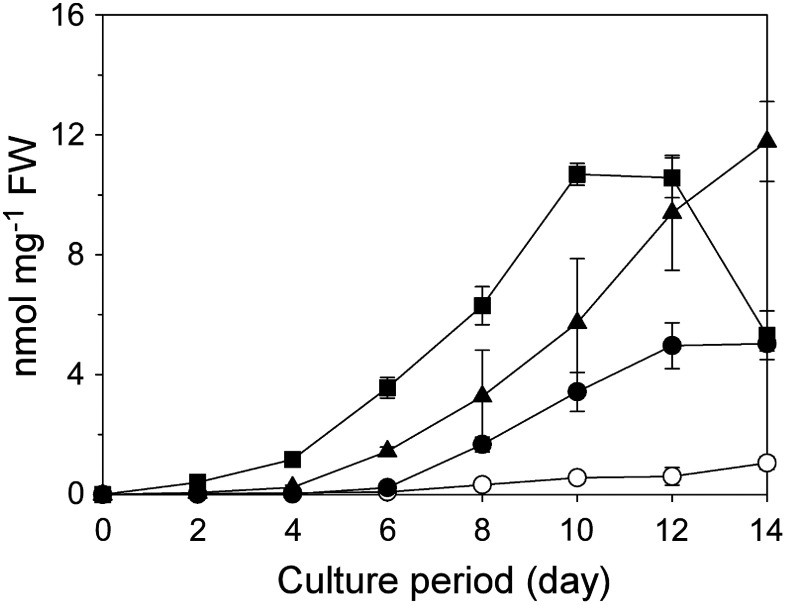
Figure 3. Time-course changes in serotonin contents in Dg suspension cells cultured in medium containing various GA_3_ concentrations. Empty circles, 0 µM (control); filled circles, 0.1 µM; filled triangles, 1 µM; filled squares, 10 µM. Data are presented as the mean±SD (*n*=3).

We examined the effect of the GA_3_ concentration on serotonin production. Specifically, Dg suspension cells were cultured for 14 days (initial cell density of 5% SCV) in 1/2 MS medium supplemented with various GA_3_ concentrations. Serotonin production varied among the GA_3_ concentrations, with 10 µM revealed as the most effective concentration for the first 10 days (Figure 3). However, after 12 days of the 10 µM GA_3_ treatment, serotonin production decreased, whereas serotonin levels continued to increase in cells treated with 1 µM GA_3_. This was likely associated with the relatively strong browning of cells after 12 days of the 10 µM GA_3_ treatment.

**Figure 4:**
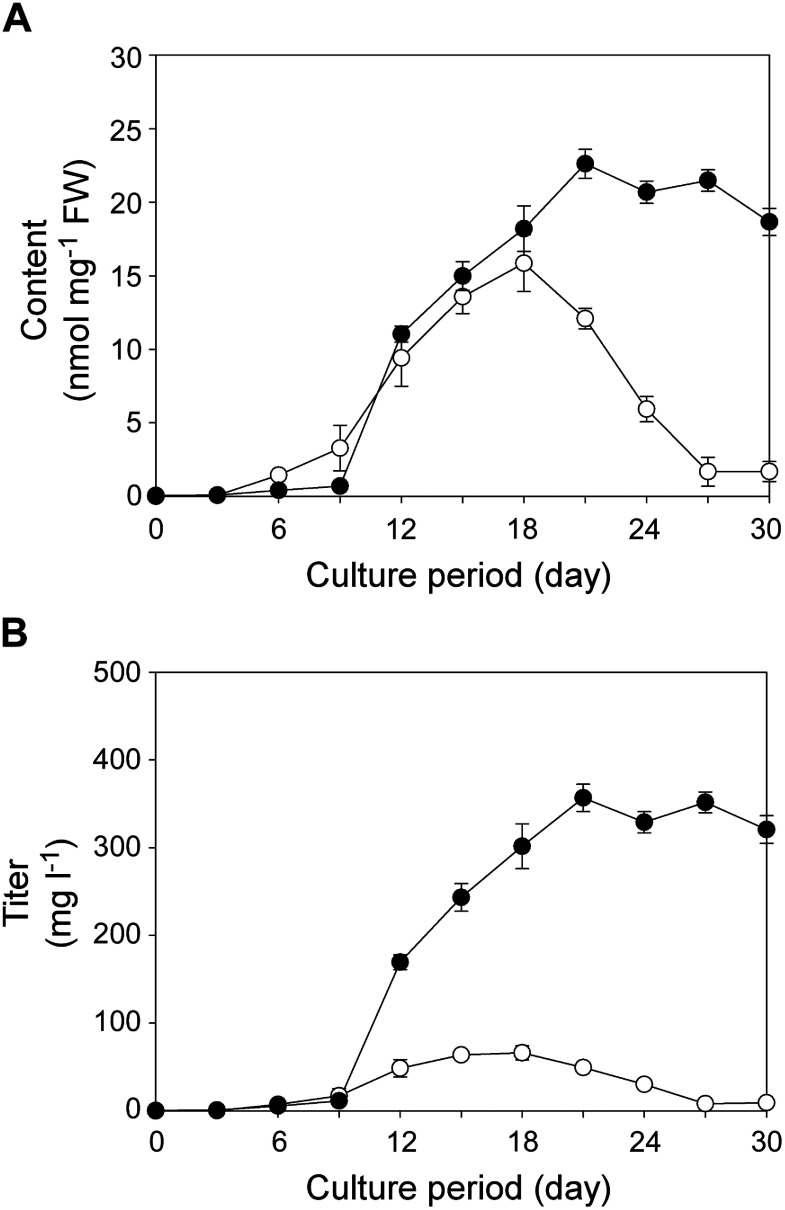
Figure 4. Effects of the initial cell density on serotonin production in Dg suspension cells cultured in medium supplemented with 1 µM GA_3_. Serotonin contents (A) and production titers (B) in cell cultures with different initial cell densities are shown. Initial cell densities were set at 5% (empty circles) and 10% (filled circles) SCVs. Data are presented as the mean±SD (*n*=3).

Because of the increase in serotonin production following the 1 µM GA_3_ treatment, we cultured Dg suspension cells for 30 days (initial cell densities of 5% or 10% SCV) in 1/2 MS medium containing 1 µM GA_3_. Similar serotonin accumulation profiles were obtained for the cultures derived from 5% and 10% SCVs for the first 18 days (Figure 4A). For the culture with an initial cell density of 5% SCV, the serotonin level peaked on day 18 (16 nmol mg^−1^ FW), but subsequently decreased to its lowest level on day 30. In contrast, for the culture with an initial cell density of 10% SCV, the serotonin content was highest on day 21 (23 nmol mg^−1^ FW). The decreased production of serotonin after 18 days for the culture with an initial cell density of 5% SCV was most likely due to the strong browning of cells, which adversely affected cell viability. However, a similar phenomenon was not observed in the culture with a higher initial cell density (10% SCV); the cell proliferation rate was unaffected by 1 µM GA_3_ (Supplementary Figure S6), while strong browning of cells was detected only on day 30. These results suggest that Dg cells metabolize GA_3_ to avoid the cytotoxic effects of excessive GA_3_. It should be noted that serotonin was hardly detectable in culture media under any culture conditions.

By multiplying the serotonin content by the cell FW at each collection time point, we calculated the serotonin titer per liter of suspension culture. The highest titer (360 mg l^−1^) was obtained on day 21 of the culture with an initial cell density of 10% SCV (Figure 4B).

### Effects of GA_3_ on Dg cell lignification

Culturing Dg cells in the presence of GA_3_ altered the physical appearance of the cells (relative to the cells cultured in the absence of GA_3_), with weak browning and hardening of cells detected during the culture period (Supplementary Figure S7). Because these are features of xylogenesis (Nomura et al. 2013; Ogita et al. 2012), we analyzed cell lignification by staining the cells with a phloroglucinol–HCl reagent. The red staining observed during the later time points for the culture treated with 1 µM GA_3_ reflected the lignification of cells (Supplementary Figure S7). Moreover, the staining of cells treated with 1 µM GA_3_ was stronger and detected earlier than the staining of cells cultured under phytohormone-free conditions. These results indicate that GA_3_ promoted the lignification of Dg cells.

Considering that Dg cells cultured in the presence of 1 µM GA_3_ accumulated serotonin, we hypothesized that the observed enhanced lignification of cells treated with 1 µM GA_3_ may be mediated by serotonin. To test this hypothesis, Dg cells were cultured in medium supplemented with various concentrations of serotonin. However, the physical appearance of cells and cell lignification profiles were generally unaffected, even after the 1,000 µM serotonin treatment (Supplementary Figure S7), indicating that serotonin is not involved in the GA_3_-induced lignification of Dg cells.

### Serotonin content in the *D. giganteus* shoot

Dg cells were generated from young edible shoot tissue of *D. giganteus*. To examine whether the biosynthetic property of serotonin in Dg cells is found only in the cultured cells or is also found in the original plant tissue, we analyzed serotonin in shoot by separating it into several parts (Supplementary Figure S8A). Serotonin was detected in all examined shoot parts (Supplementary Figure S8B), but the serotonin content was highest (0.72 nmol mg^−1^ FW) in the middle part of the shoot. Serotonin was also detected in the peel, with higher contents in the outer peel (0.42 nmol mg^−1^ FW) than in the inner peel (0.040 nmol mg^−1^ FW). Serotonin biosynthesis was almost undetectable in Dg cells cultured under GA_3_-free conditions, but it was activated in cells cultured in the presence of GA_3_. The highest serotonin content (23 nmol mg^−1^ FW) in Dg cells in this study (Figure 4A) was approximately 30-fold higher than the highest serotonin content in the shoot (0.72 nmol mg^−1^ FW).

## Discussion

Rational metabolic-flow switching is a metabolic engineering strategy that we proposed previously for the efficient production of exogenous secondary metabolites using cultured plant cells in which a highly active inherent metabolic pathway is redirected to a pathway producing related exogenous compounds (Nomura et al. 2018). Through a series of proof-of-concept studies, we demonstrated that Pn bamboo cells are suitable for the bioproduction of phenylpropanoid-derived compounds via rational metabolic-flow switching (Kitaoka et al. 2020, 2021; Nomura et al. 2018; Ube et al. 2024). In the current study, to identify cultured plant cells appropriate for the bioproduction of other types of secondary metabolites, we analyzed the metabolic properties of Dg and Db cells (i.e., newly generated cultured cells of tropical bamboo species) treated with phytohormones. According to the study findings, Dg cells can produce high levels of serotonin in response to GA_3_. GA_3_ is reported to function as an effective elicitor for production of some secondary metabolites in plants and cultured tissues and cells, such as phenylpropanoid- and terpenoid-related compounds in grapevine (*Vitis vinifera*) plant, *Echinacea purpurea* hairy roots, and *Artemisia absinthium* suspension cultures (Abbasi et al. 2012; Ali et al. 2015; Murcia et al. 2017). To our knowledge, induction of the biosynthesis of tryptophan-derived indolic compound serotonin by GA_3_ treatment has not been reported previously.

In plants, serotonin is generally biosynthesized from tryptophan via tryptamine (Ramakrishna et al. 2011). It is likely that the expression of genes encoding biosynthetic enzymes (tryptophan decarboxylase and tryptamine 5-hydroxylase) is highly activated by a GA_3_ treatment. Introducing exogenous gene(s) encoding enzyme(s) affecting tryptophan, tryptamine, or serotonin contents to Dg cells may facilitate the production of exogenous indolic compounds. For example, gramine (3-[dimethylaminomethyl]indole), a simple tryptophan-derived alkaloid involved in the chemical defense of barley (*Hordeum* spp.), may be produced using transgenic Dg cells. The gramine biosynthetic pathway involves two specific enzymes, CYP76M57 and *N*-methyltransferase (Dias et al. 2024; Ishikawa et al. 2024; Larsson et al. 2006), with the former catalyzing the conversion of tryptophan to 3-(aminomethyl)indole and the latter catalyzing two sequential *N*-methylations of the amino group of 3-(aminomethyl)indole to form gramine. Gramine bioproduction may be achieved by simply culturing transgenic Dg cells overexpressing the genes encoding the two biosynthetic enzymes under optimized GA_3_ conditions. As the peak serotonin content in the present study (23 nmol mg^−1^ FW≈4 mg g^−1^ FW) is several orders of magnitude higher than the serotonin contents reported in other plant species (Ramakrishna et al. 2011), the production of large amounts of gramine will be achieved by redirecting this metabolic property to the gramine biosynthesis in transgenic Dg cells. The use of eucaryotic Dg cells as production host, unlike procaryotic host such as *Escherichia coli*, is also favorable for correct expression and localization of the membrane-bound CYP76M57, the key enzyme to redirect the biosynthesis of serotonin to the biosynthesis of gramine.

Considering the metabolic activities of GA_3_-treated Dg cells, hydroxycinnamic acid amides of serotonin, such as *N*-*p*-coumaroylserotonin and *N*-feruloylserotonin, may be other compounds that can be produced using transgenic Dg cells. Dg cells cultured in the presence of GA_3_ exhibited increased lignification, indicating that monolignol biosynthesis is activated along with serotonin biosynthesis. Because *N*-*p*-coumaroylserotonin and *N*-feruloylserotonin are biosynthesized via the condensation of serotonin and *p*-coumaroyl- and feruloyl-CoAs (i.e., intermediates of monolignol biosynthesis) in reactions catalyzed by serotonin *N*-hydroxycinnamoyltransferase (Jang et al. 2004), introducing and overexpressing the gene encoding this enzyme in Dg cells may lead to the efficient bioproduction of these hydroxycinnamic acid amides, which are useful compounds with antioxidative and antifungal effects (Hotta et al. 2002; Tanaka et al. 2003).

When serotonin is used as a substrate by an exogenous enzyme to produce serotonin derivatives, increasing the available serotonin content is crucial for optimizing the production of target compounds. Regarding this point, an intriguing finding was reported for a previous study. Specifically, in rice leaves infected with a pathogenic fungus, serotonin accumulated and was subsequently incorporated into cell wall fractions via peroxidase-catalyzed oxidative polymerization, thereby increasing cell wall strength (Ishihara et al. 2008). In fact, when Dg cells were treated with 1,000 µM serotonin, serotonin was almost undetectable in the culture medium after 1 week, while the serotonin content in the treated cells was similar to that in the control cells. These results strongly suggest that serotonin was incorporated in cells and then metabolized to undetectable forms (most likely polymerized constituents of cell wall fractions). Therefore, inhibiting the peroxidase activity for serotonin polymerization using additives, such as salicylhydroxamic acid and sodium L-ascorbate (Okazaki et al. 2004), may increase the abundance of soluble serotonin in cells to be used as the substrate for exogenous enzymes. We are currently verifying whether this approach facilitates the elevated levels of soluble serotonin and serotonin-derived exogenous metabolites in the wild-type Dg cells and the above-mentioned transgenic Dg cells, respectively.

In the present study, serotonin in Dg cells was the sole secondary metabolite that was markedly induced by treatments with various phytohormones. Nevertheless, in addition to Dg cells, Db cells should also have cryptic secondary metabolic properties. Recently, we demonstrated that epigenetic modifiers, including histone deacetylase and DNA methyltransferase inhibitors, can activate the biosynthesis of cryptic secondary metabolites in cultured plant cells (Nomura and Kato 2025; Nomura et al. 2021, 2022). This may have implications for developing cell culture systems that continuously produce substantial amounts of target secondary metabolites as well as for mining novel compounds. This approach may be useful for activating cryptic secondary metabolite-related cellular processes that are not activated by phytohormones. Moreover, the synergetic effects of epigenetic modifiers and phytohormones should also be investigated. Exploiting the metabolic properties of various cultured plant cells through such approaches can increase the number of viable cell type–culture condition combinations for specific target compounds produced via rational metabolic-flow switching.

## Abbreviations

Db: *Dendrocalamus brandisii*
Dg: *Dendrocalamus giganteus*
GA_3_: gibberellin A_3_
mMS: modified Murashige and Skoog
MS: Murashige and Skoog
Pn: *Phyllostachys nigra*
SCV: sedimented cell volume

## Description of supplementary files

Supplementary Figure S1. *D. giganteus* (Dg) (A) and *D. brandisii* (Db) (B) bamboo shoots.

Supplementary Figure S2. *D. giganteus* (Dg) callus induction.

Supplementary Figure S3. HR-ESI-TOF-MS spectrum of serotonin (acetic acid salt, 1) isolated in this study.

Supplementary Figure S4. NMR spectra of serotonin (acetic acid salt, 1) isolated in this study.

Supplementary Figure S5. Colorimetric analyses of serotonin (acetic acid salt, 1) isolated in this study indicating the presence of a primary amino group (A), indole skeleton (B), and phenolic hydroxy group (C).

Supplementary Figure S6. Effects of the initial cell density on the proliferation of Dg suspension cells cultured in the presence of 1 µM GA_3_.

Supplementary Figure S7. Lignification profiles of Dg suspension cells cultured in the presence of GA_3_ or serotonin.

Supplementary Figure S8. Serotonin contents in different parts of a young *D. giganteus* shoot.
