# Supplementary material for: Induced accumulation of serotonin in gibberellin A3-treated suspension cells of giant bamboo (Dendrocalamus giganteus)
Source: Plant Biotechnol (Tokyo). 2025 Mar 25;42(1):65–72. doi: 10.5511/plantbiotechnology.25.0113a (PMC12622899; doi:10.5511/plantbiotechnology.25.0113a)
Supplement: Supplementary Data [file plantbiotechnology-42-1-25.0113a-s001.pdf]

Supplementary Information

for:

Induced accumulation of serotonin in gibberellin A_3-treated suspension cells of giant bamboo (*Dendrocalamus giganteus*)

Taiji Nomura[1,*], Shinjiro Ogita[2], Yasuo Kato[1]

[1]*Biotechnology Research Center and Department of Biotechnology, Toyama Prefectural University, 5180 Kurokawa, Imizu, Toyama 939-0398, Japan*

[2]*Department of Development of Local Resources, Faculty of Bioresource Sciences, Prefectural University of Hiroshima, 5562 Nanatsukacho, Shobara, Hiroshima 727-0023, Japan*

[*]Corresponding author e-mail: tnomura@pu-toyama.ac.jp

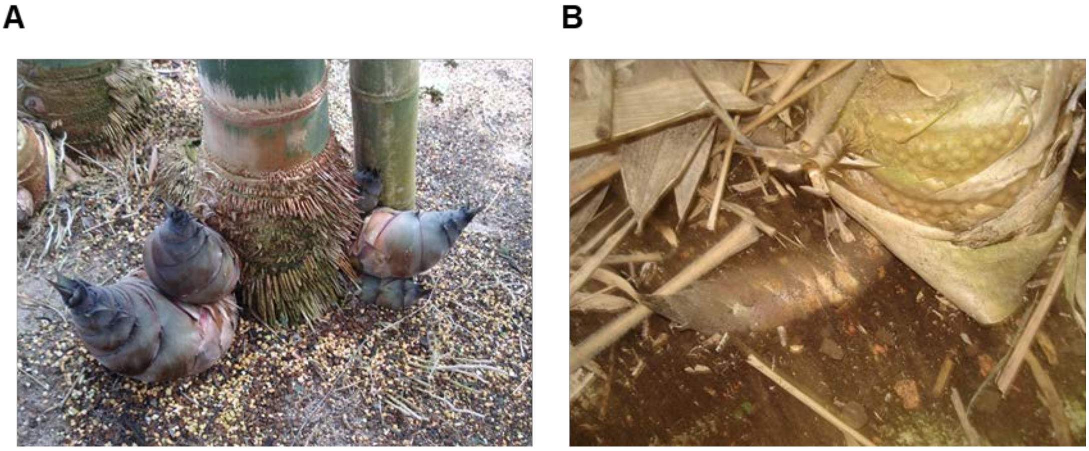

Supplementary Figure S1. *D. giganteus* (Dg) (A) and *D. brandisii* (Db) (B) bamboo shoots.

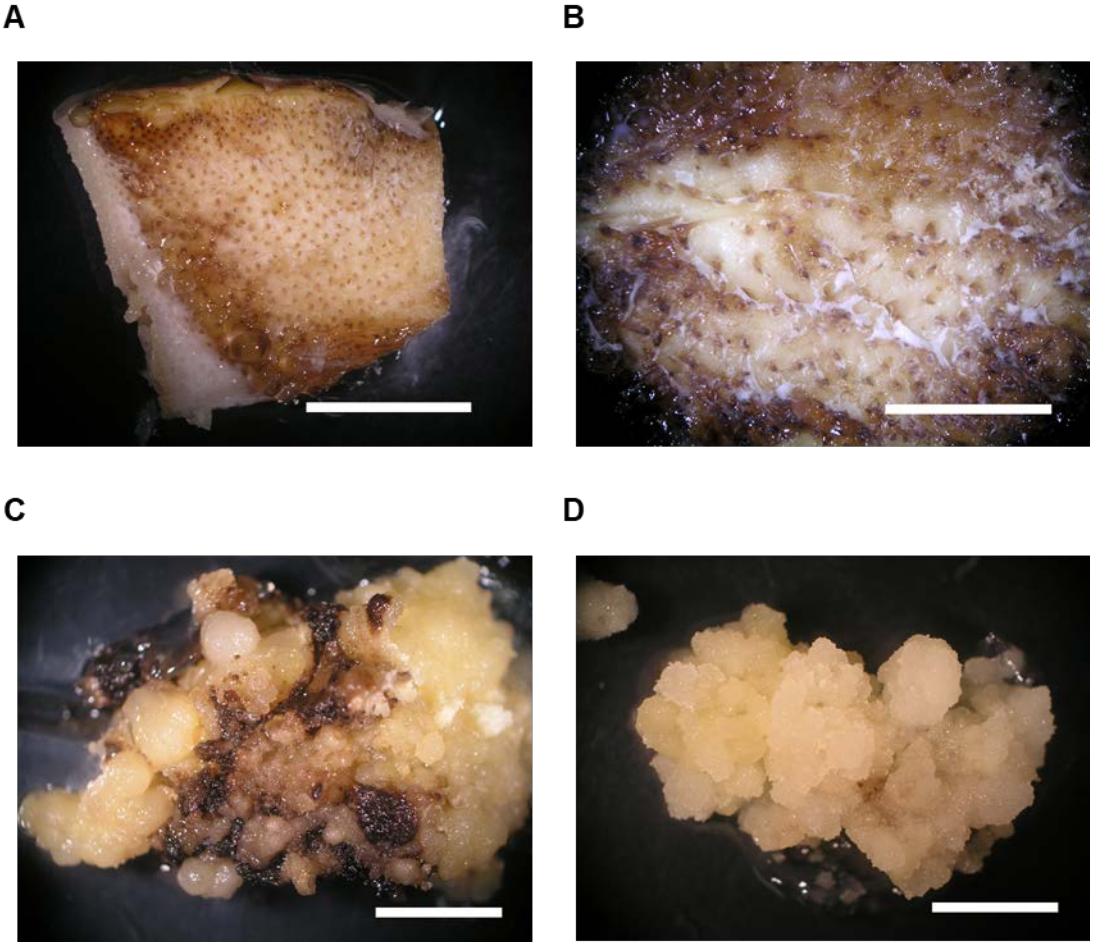

Supplementary Figure S2. *D. giganteus* (Dg) callus induction. Some shoot segments increased in size in 2 weeks (A). The surface of shoot segments swelled and produced a few calli in 4 weeks (B). Negative proliferation of hard partially necrotic calli in 6 months (C). Positive proliferation of soft calli with uniform features in 6 months (D). Scale bars represent 1 cm (A, C, and D) and 0.5 cm (B). Representative images of Dg callus induction are presented; *D. brandisii* callus induction proceeded in a similar manner.

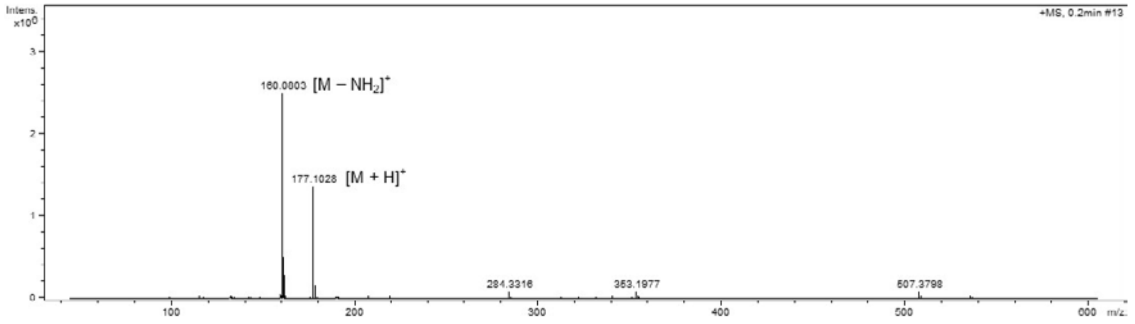

Supplementary Figure S3. HR-ESI-TOF-MS spectrum of serotonin (acetic acid salt, 1) isolated in this study.

A

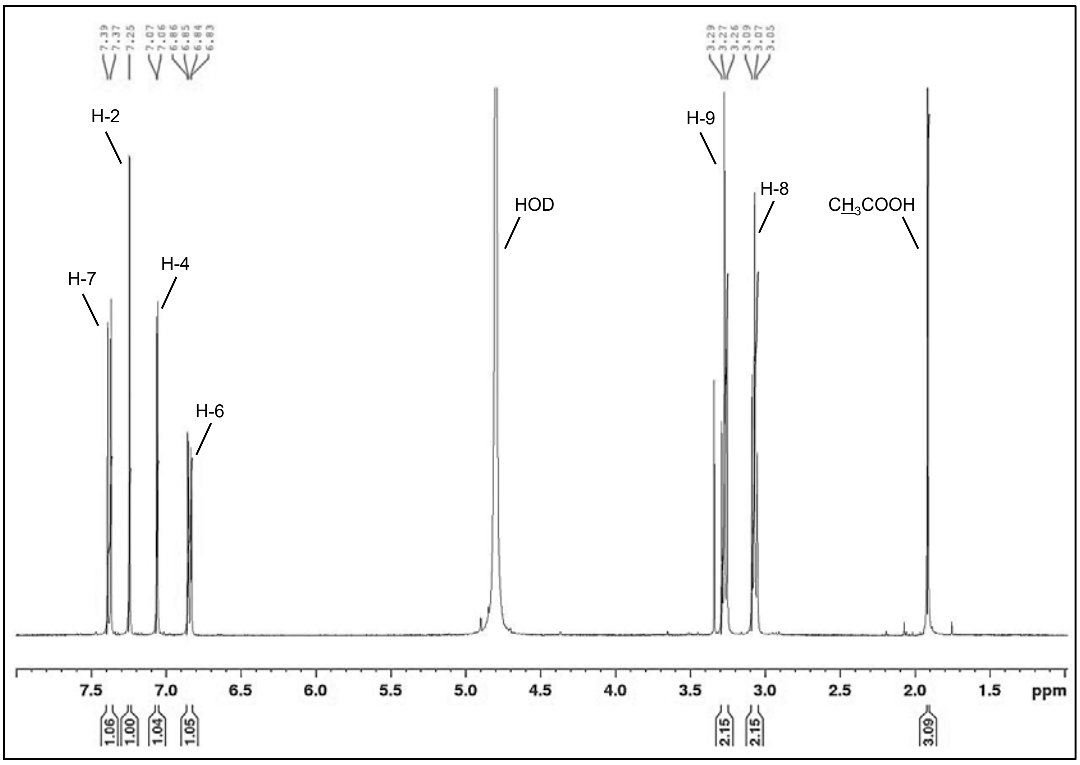

B

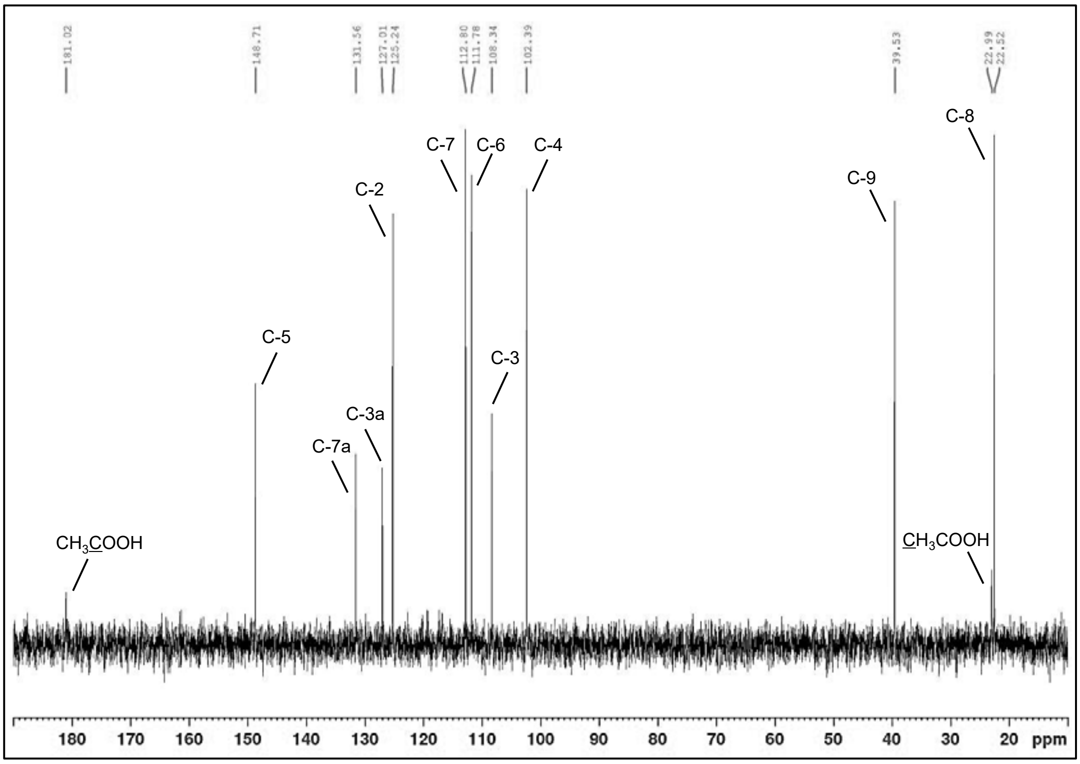

Supplementary Figure S4. NMR spectra of serotonin (acetic acid salt, 1) isolated in this study. (A) ^{1}H-NMR spectrum (400 MHz, D_2O). (B) ^{13}C-NMR spectrum (100 MHz, D_2O).

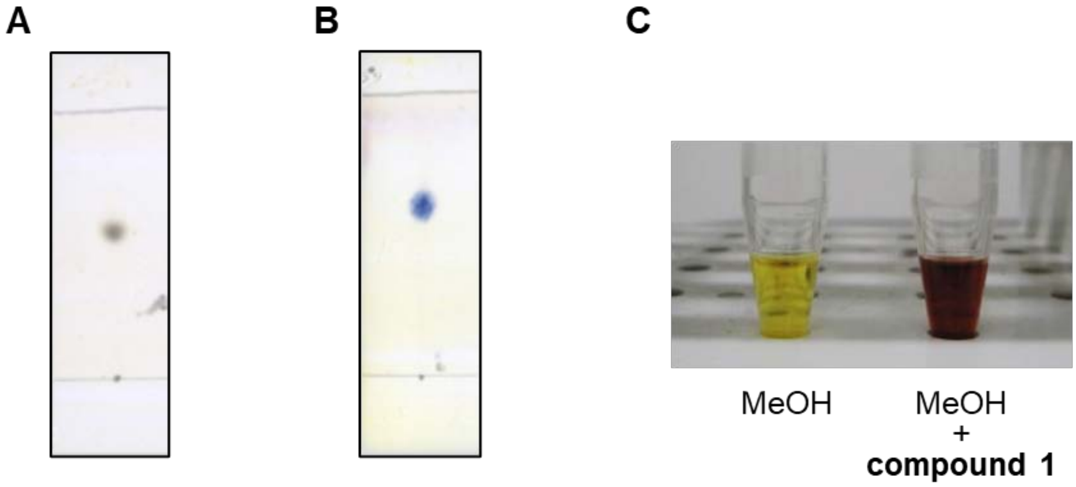

Supplementary Figure S5. Colorimetric analyses of serotonin (acetic acid salt, 1) isolated in this study indicating the presence of a primary amino group (A), indole skeleton (B), and phenolic hydroxy group (C).

Compound 1 spotted on TLC silica gel 60 F_{254} (Merck, Darmstadt, Germany) was developed using 2-propanol:ammonia aq. (28%):water = 8:1:1 as the solvent and then detected by spraying the plate with a ninhydrin reagent (A) or Ehrlich's reagent (B) and then heating. MeOH solution of compound 1 (10 mM) was mixed with an equal volume of 2.5% (w/v) $FeCl_3 \cdot 6H_2O$ (C).

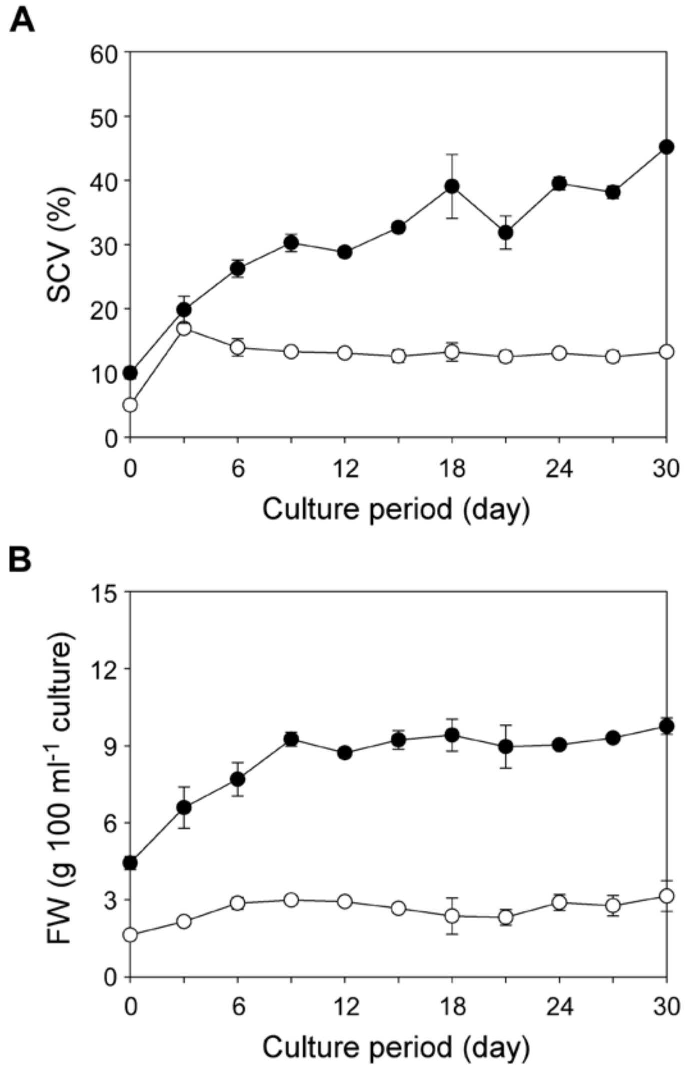

Supplementary Figure S6. Effects of the initial cell density on the proliferation of Dg suspension cells cultured in the presence of 1 μM GA_3.

SCV (A) and FW (B) of cell cultures with different initial cell densities are shown. Initial cell densities were set at 5% (empty circles) and 10% (filled circles) SCVs. Data are presented as the mean ± SD (n = 3).

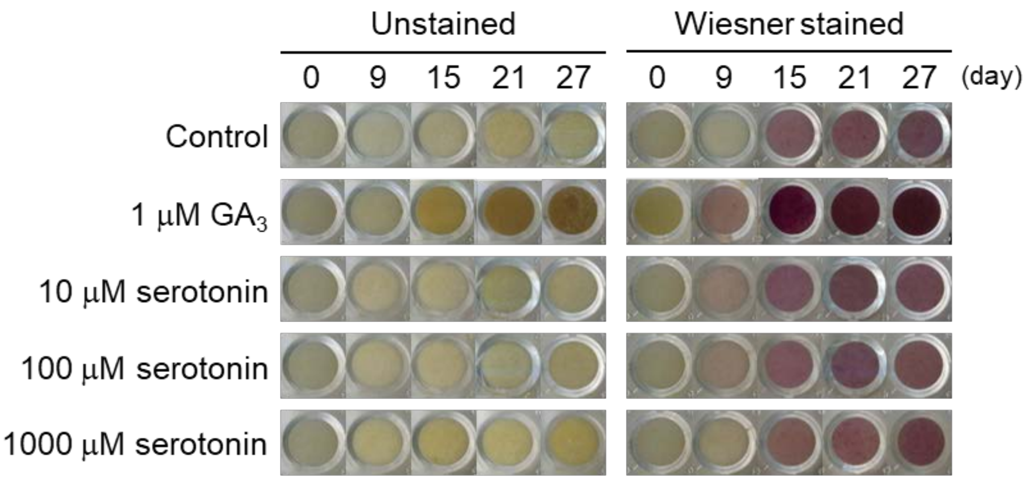

Supplementary Figure S7. Lignification profiles of Dg suspension cells cultured in the presence of GA_3 or serotonin.

A portion of the cells collected at each time point were fixed and stained with a phloroglucinol–HCl (Wiesner) reagent in a 96-well microplate as described previously (Ogita et al. 2012).

A

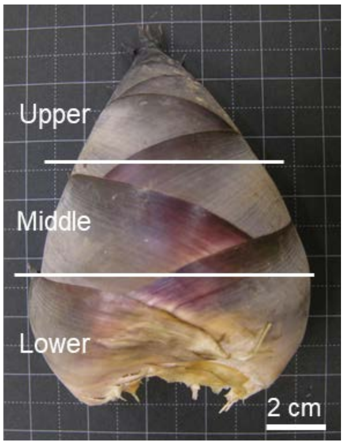

B

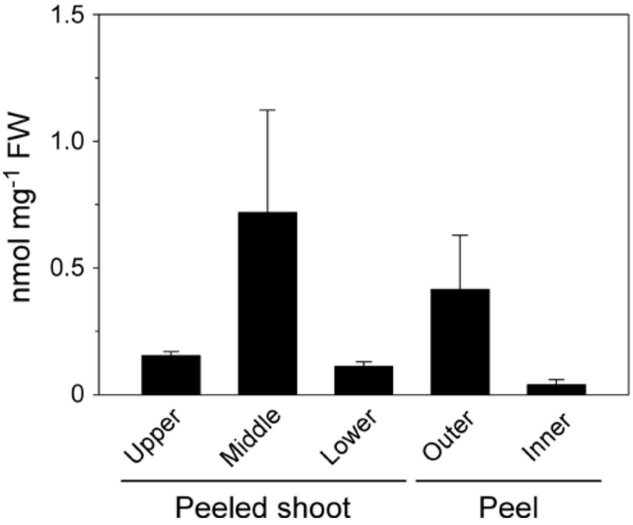

Supplementary Figure S8. Serotonin contents in different parts of a young *D. giganteus* shoot.

(A) *D. giganteus* shoot used for the analysis. The shoot was peeled and divided into three parts (upper, middle, and lower). The peel was separated into the outer (hard, fibrous) and inner (soft) parts. (B) Serotonin contents in each shoot part. Approximately 100 mg tissues were frozen in liquid nitrogen and then ground to a fine powder using a mortar and pestle for the subsequent extraction using 10 volumes of 50% (v/v) MeOH containing 2% (v/v) acetic acid. After each extract was centrifuged (21,500 × *g*, 10 min, 4°C), the supernatant was appropriately diluted in water and subjected to a reversed-phase HPLC analysis as described in the main text. Data are presented as the mean ± SD (n = 3).

Reference for Supplementary Information

Ogita S, Ohki S, Nomura T, Kato Y (2012) A β-glucosidase activity potentially involved in cell division and wall development of *Phyllostachys* bamboo suspension cells. *Am J Plant Sci* 3: 1066–1072
